# Optical coherence tomography for broad spectrum of thyroid carcinoma: a qualitative and quantitative analysis

**DOI:** 10.1007/s13534-026-00582-z

**Published:** 2026-04-18

**Authors:** Woojin Lee, Soonyong Kwon, Hyeong Soo Nam, Hongki Yoo, Jae Yeon Seok

**Affiliations:** 1 Department of Mechanical Engineering, Korea Advanced Institute of Science and Technology (KAIST), 291 Daehak-ro, Yuseong-gu, Daejeon, 34141 Republic of Korea; 2 Department of Pathology, Yongin Severance Hospital, Yonsei University College of Medicine, 363, Dongbaekjukjeon-daero, Giheung-gu, Yongin-si, Gyeonggi-do 16995 Republic of Korea

**Keywords:** Thyroid, Carcinoma, Pathology, Optical coherence tomography, Real-time, Label-free

## Abstract

Optical coherence tomography (OCT) is a non-invasive imaging modality providing real-time micrometer-scale resolution without conventional tissue processing. This study evaluated the diagnostic potential of OCT for distinguishing normal thyroid tissue from a broad spectrum of thyroid carcinomas in a pathological context. OCT imaging was performed on formalin-fixed thyroid specimens from 41 cases, including papillary (PTC, *n* = 11), follicular (FTC, *n* = 6), oncocytic (OCA, *n* = 3), anaplastic (ATC, *n* = 9), and medullary thyroid carcinoma (MTC, *n* = 12), with corresponding hematoxylin and eosin sections for correlation. Quantitative parameters were developed or adopted, including surface irregularity, penetration depth, tissue brightness, and gray-level co-occurrence matrix texture features. Qualitative analysis demonstrated visualization of tumor-specific architectural patterns, while three-dimensional reconstruction enabled depth-resolved assessment of capsular invasion and peri-capsular vascular structures. All quantitative metrics significantly differentiated carcinoma from normal tissue and showed discriminative capability across carcinoma subtypes. These findings highlight OCT as a high-resolution, label-free modality with utility in thyroid cancer diagnosis.

**Supplementary Information:**

The online version contains supplementary material available at 10.1007/s13534-026-00582-z.

## Introduction

Thyroid cancer, the most common endocrine malignancy, accounts for approximately 4.7% of newly diagnosed cancers worldwide, ranking seventh overall and fifth among women [1]. Among thyroid cancers, papillary thyroid carcinoma (PTC) represents 80–85%, followed by follicular thyroid carcinoma (FTC), oncocytic carcinoma (OCA), medullary thyroid carcinoma (MTC), and anaplastic thyroid carcinoma (ATC) [2]. Differentiated thyroid carcinomas (DTCs; PTC, FTC, OCA) generally have excellent prognoses and are managed with thyroidectomy, lymph node dissection, and radioactive iodine [3, 4]. In contrast, ATC is highly aggressive [3, 5], and MTC, a neuroendocrine tumor from parafollicular C cells, has a hereditary component requiring early detection and genetic screening [2].

Ultrasonography (US) with fine-needle aspiration (FNA) cytology is the gold standard for preoperative evaluation [3]. Yet up to 30% of FNAs are indeterminate, often leading to diagnostic surgery or intraoperative frozen section (FS) [6]. Approximately 75% of such surgeries prove benign, indicating unnecessary interventions. Molecular testing is recommended for indeterminate FNA but limited by cost and infrastructure.

FS offers rapid assessment but has limitations—tissue destruction, difficulty with calcified/small nodules, and frozen artifacts impairing nuclear evaluation—raising questions about its value [7, 8]. Furthermore, intraoperative parathyroid gland identification, critical for preventing hypoparathyroidism, may paradoxically compromise preservation when FS is used.

Optical coherence tomography (OCT) is a non-invasive, label-free, high-resolution modality that detects backscattered light interferometrically employing a broadband light source [9, 10]. The depth profile of the sample is obtained by subjecting Fourier transform to the interference signal, allowing for precise depth-resolved imaging and three-dimensional (3D) imaging potential. Leveraging these capabilities and biosafety, OCT is widely adopted in ophthalmology [11], cardiology [12, 13], dermatology [14], and gastroenterology [15].

Interest is growing in applying OCT to thyroid cancer diagnostics [16–23] and parathyroid identification [18, 19, 23–28]. Prior studies compared OCT with histopathology, mainly targeting normal thyroid gland (NT), parathyroid gland, fat, and lymph node tissue [19, 23, 27, 28], or benign thyroid nodules/lesions (multinodular goiter, lymphocytic thyroiditis, follicular adenoma etc.) [16, 17, 19–23]. Malignancy analyses were limited to small cohorts of PTCs [16–18, 20, 22, 23], FTC [23], or MTC [16], and qualitative.

This study evaluated OCT for thyroid cancer diagnosis across a broad carcinoma spectrum. We introduced novel quantitative parameters—surface irregularity, penetration depth, and tissue brightness—and applied gray-level co-occurrence matrix (GLCM) texture analysis to differentiate normal and malignant thyroid tissues.

## Results

**Fig. 1:**
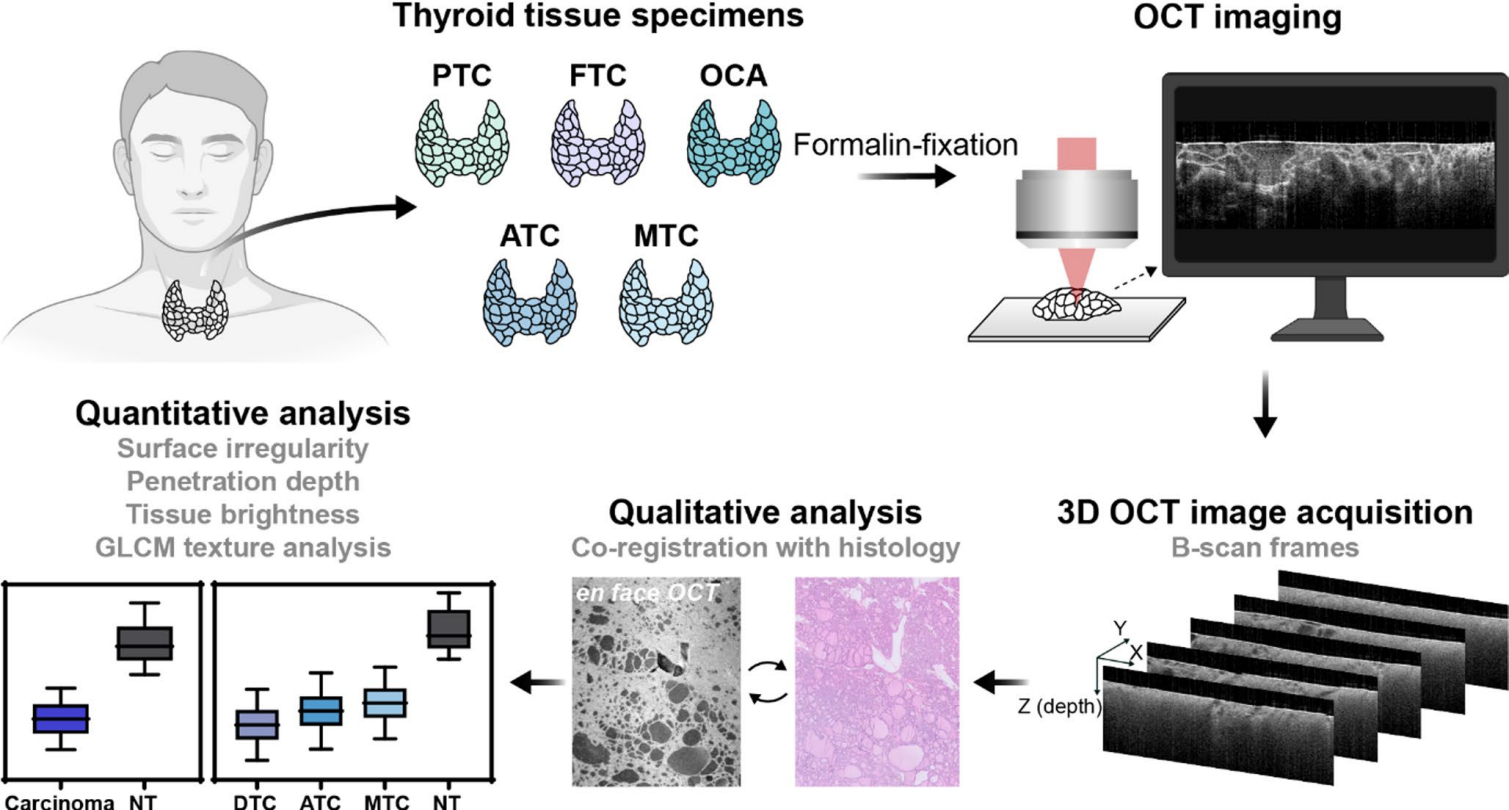
Overview of the OCT-based pathological study for thyroid carcinoma. A schematic diagram presents the overall workflow of the OCT-based pathological analysis in thyroid carcinoma. Resected human thyroid specimens, fixed in formalin, were imaged employing a benchtop swept-source OCT system. Three-dimensional OCT datasets were acquired from pre-selected regions of interests (ROIs), and en face OCT images were subsequently reconstructed. These en face images were co-registered with corresponding histological sections. Representative ROIs from each case were then selected for detailed pathological interpretation and quantitative analysis

Figure 1 summarizes the overall study design and pathological evaluation workflow. To investigate the diagnostic potential of OCT across a broad spectrum of thyroid carcinoma, OCT imaging and quantitative analysis were performed on surgically resected thyroid cancer specimens from 41 patients, including PTC (*n* = 11), FTC (*n* = 6), OCA (*n* = 3), ATC (*n* = 9), and MTC (*n* = 12). Representative tissue sections for OCT imaging were selected by a pathologist, and paraffin blocks were reciprocally processed to obtain formalin-fixed specimens suitable for direct comparison with histologic sections.

OCT imaging was performed using a benchtop swept-source (SS)-OCT system, enabling acquisition of volumetric datasets for en face visualization and 3D reconstruction of tissue architecture. Through direct comparison with corresponding histologic sections, OCT findings were used to identify characteristic architectural and compositional signatures of thyroid carcinoma and to evaluate depth resolved morphological changes using the volumetric imaging capability of OCT. For quantitative validation of OCT based discrimination across the broad spectrum of thyroid carcinoma, we evaluated whether OCT images capture measurable differences between NT and carcinoma, as well as among distinct carcinoma subtypes. OCT derived parameters reflecting tissue morphological characteristics, including surface irregularity, penetration depth, and tissue brightness, were defined and analyzed. In addition, texture features derived from GLCM analysis were evaluated to capture cellular level differences within tissue microstructure across thyroid carcinoma subtypes. Detailed descriptions are provided in the “Methods” section.

**Fig. 2:**
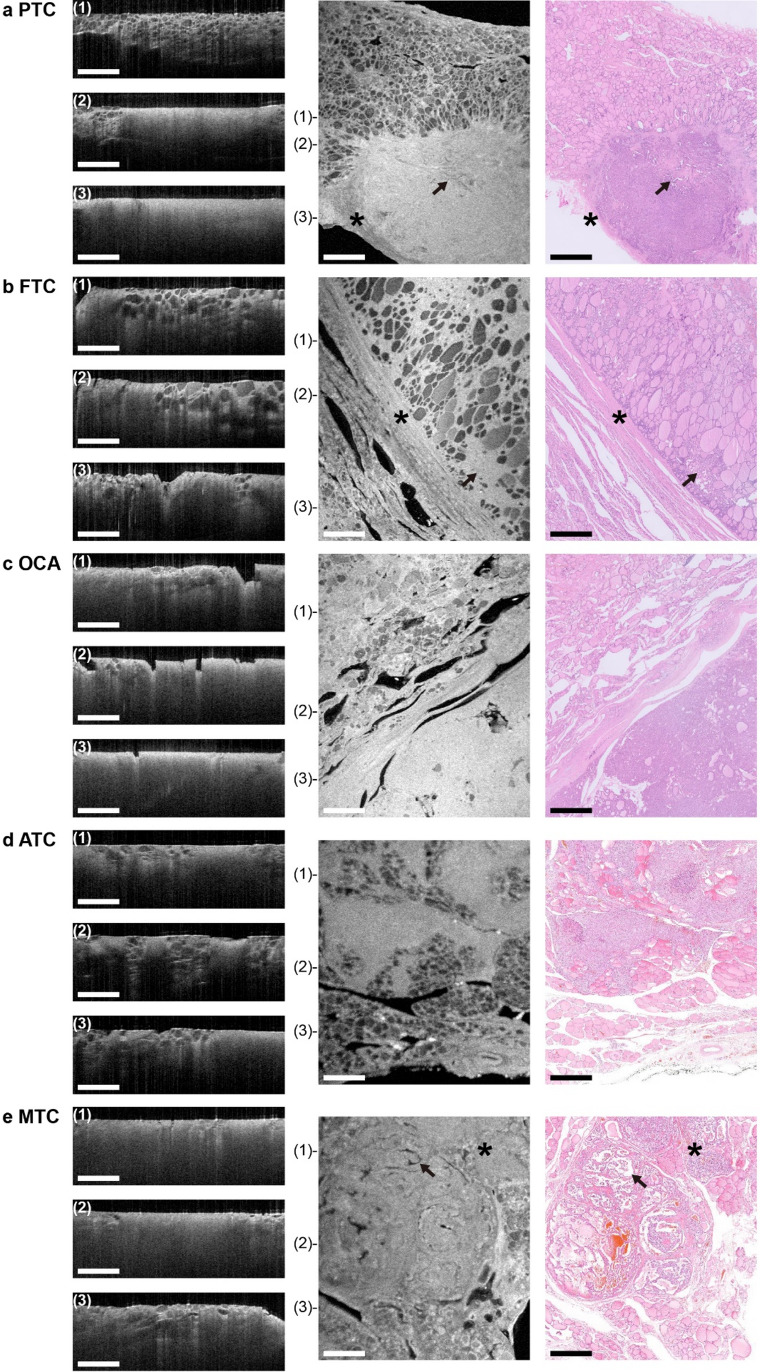
Representative OCT images of each thyroid carcinoma group. The B-scan, en face OCT image, and the corresponding histological section are displayed. The position of the B-scan within the en face plane is indicated at the top left of each B-scan image and adjacent to the corresponding en face image. **a** PTC. The PTC shows compact papillary growth with slit-like space (arrow) in en face OCT image. The collagen fiber of tumor fibrous capsule shows its own alignment direction (asterisk). **b** FTC. The intra-tumoral follicular structure is evident in FTC except the microfollicular area (arrow). The elongated collagen fiber of tumor fibrous capsule is identified with multilayering appearance (asterisk). **c** OCA. The microfollicular pattern of OCA is identified as a relatively homogeneous solid feature with high signal intensity in OCT images. **d** ATC. The infiltrative feature of ATC is demonstrated replacing normal follicular structure. The signal intensity is relatively low and the penetration depth is short comparing to the adjacent normal thyroid parenchyma. **e** MTC. Diverse histologic pattern in MTC is identified (arrow: papillary, asterisk: conventional). All scale bars, 1 mm

### Qualitative analysis results: en face OCT image correlation with H&E pathologic images

Representative OCT images of the five thyroid carcinoma groups, namely PTC, FTC, OCA, ATC, and MTC, are presented in Fig. 2. Each case includes B-scan images, en face reconstructions, and corresponding histological sections to enable direct morphological correlation.

#### PTC

In en face OCT of conventional PTC, the tumor showed bright reflective signal and elongated papillary structure with slit-like spaces in a vaguely solid mass (Fig. 2a). When follicular pattern existed, the colloid within intra-tumoral follicles resembled normal thyroid colloid (Fig. 2a). The fibrous capsule showed collagen fibers oriented perpendicular to tumor growth, and the brightness varied with tissue density (Fig. 2a). Capsular invasion was identified by a bright tumor bud disrupting the moderately dark fibrous capsule. The psammoma bodies, lamellated small round calcification, appeared as highly bright round spots (Supplementary Fig. 1a). When densely packed, papillary architecture was indistinct, appearing as internal coarseness within solid mass (Supplementary Fig. 1b). Solid/trabecular PTC showed relatively homogeneous solid sheet separated by thin fibrous septa (Supplementary Fig. 1c). In tall cell PTC, elongated papillae with tram-track appearance were identified (Supplementary Fig. 1d). Hobnail features were not clearly demonstrated, likely due to resolution limits.

#### FTC

In Fig. 2b, the B-scan OCT image showed FTC mass filled with intra-tumoral follicles with fibrous capsule. The pushing effect of tumor is evident in the en face OCT image, flattened follicles in the adjacent thyroid parenchyma pressed by the mass. Normo- or macro-follicular patterns were readily identified, while microfollicular pattern appeared solid in OCT. The intra-tumoral follicular structure was well delineated in terms of shape, size, density, and growth direction. The tumor cells were relatively bright compared to the adjacent NT parenchyma (Fig. 2b). Thick fibrous capsules appeared multilayered (Fig. 2b). Calcified stroma was highly reflective.

#### OCA

The OCA was composed of oncocytic cells, having abundant eosinophilic granular cytoplasm filled with mitochondria. In this cohort, the OCA showed mainly microfollicular pattern growth. The architectural features were similar to FTC (Fig. 2c), but the tumor cells exhibited relatively higher signal intensity compared to those of FTC (Fig. 2c).

#### ATC

The ATC showed highly infiltrative tumor border (Fig. 2d). The undifferentiated tumor cells exhibited a pattern-less growth pattern, frequently admixed with inflammatory cells. Giant cell-rich ATC showed multiple infiltrative foci with coarse granularity (Fig. 2d). Squamous carcinoma pattern showed irregular sheets of tumor cells with brighter reflective signal than dark inflammatory cell-rich stroma (Supplementary Fig. 2a). The geographic necrosis was frequently observed in ATC, where the reflective signal was low and appeared slightly dark (Supplementary Fig. 2b).

**Fig. 3:**
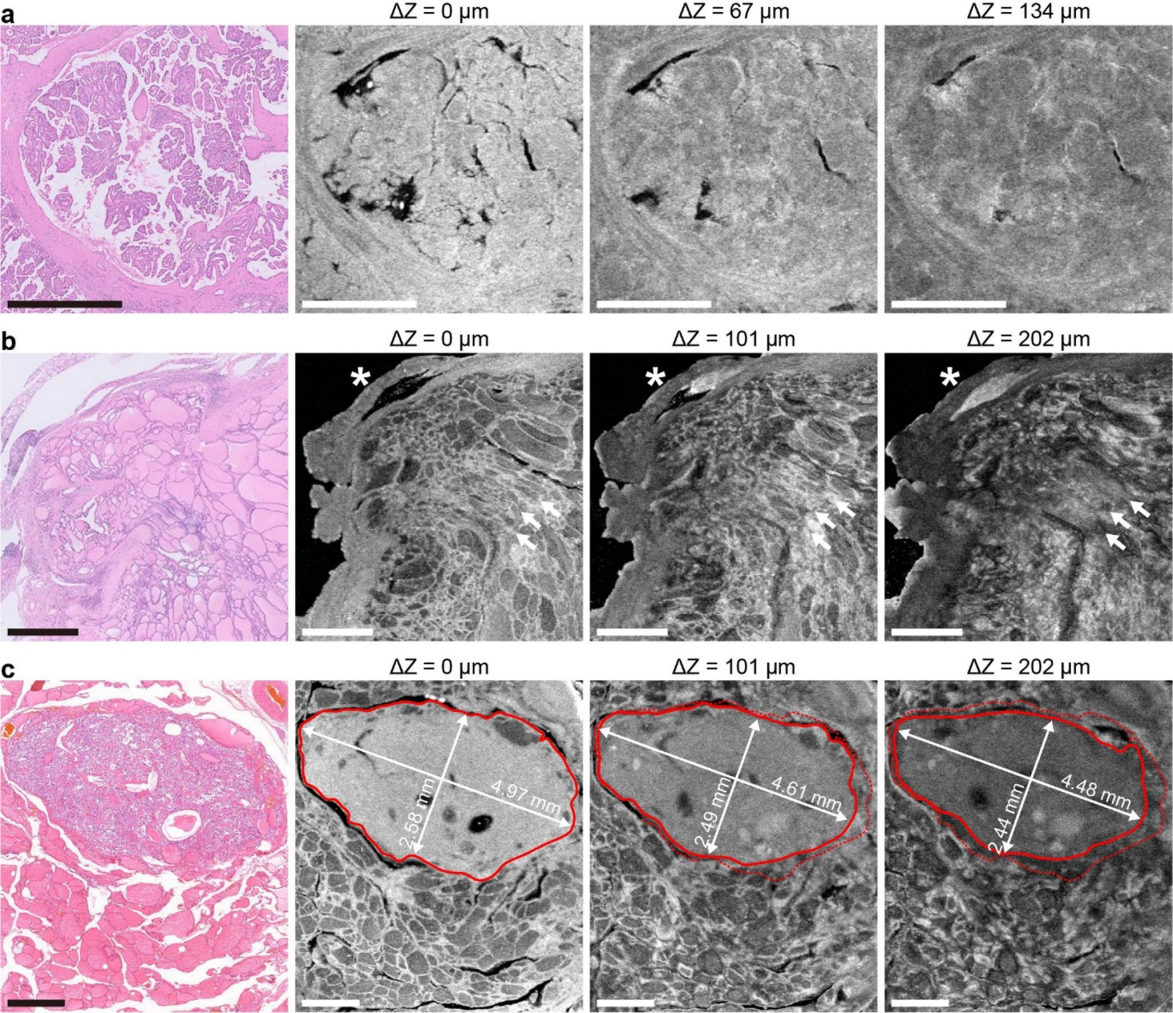
Depth-dependent structural variations visualized by OCT imaging. The depth-specific architectural alteration is revealed by OCT imaging. The corresponding histological section is shown alongside the en face OCT image. The image on the left corresponds to the original en face plane, with subsequent frames arranged toward the right with increasing depth at same intervals. **a** Papillary architecture in PTC. Note the retained high signal intensity of the outer layer of papillary structure, in which the carcinoma cells align, until greater depths. **b** The capsular invasion focus of FTC. The intra-tumoral follicular structure appears elongated perpendicular to the fibrous tumor capsule representing the pushing force vector (arrows). The intracapsular vascular space is progressively occupied by fibrinous material (asterisk). **c** The OCT images at successive depth intervals demonstrate decreasing tumor dimension in this medullary thyroid carcinoma case. This indicates the three-dimensional ovoid nature. The red line indicates the tumor outline at each imaging plane, with the red dotted line representing the outline from the first plane for comparison. Tumor boundaries were manually delineated with reference to the corresponding histological sections and reviewed by a certified pathologist (JYS). All scale bars, 1 mm

#### MTC

The diverse architectural pattern of MTC was well identified; conventional, papillary, solid, and cystic (Fig. 2e and Supplementary Fig. 2c). The stromal amyloid deposition area showed relatively brighter appearance with high reflective signal (Supplementary Fig. 2d).

#### Non-neoplastic tissues

The follicular epithelial cells appeared as thin rims of round structures with moderately bright signals (Supplementary Fig. 3a). The intra-follicular colloid showed a dark homogeneous texture, with brightness varying according to density (Supplementary Fig. 3a). Follicular nodular disease displayed hyperplastic follicles forming nodules, with variable size and shape—micro-, normo-, macro-follicular and occasional cystic change (Supplementary Fig. 3b).

In lymphocytic thyroiditis, lymphoid follicles appeared granular, round to ovoid with fuzzy outlines (Supplementary Fig. 3c). Mild infiltration and fibrosis caused parenchymal haziness with follicular remnants (Supplementary Fig. 3b).

The normal parathyroid parenchyma was slightly bright with occasional dark vacuoles (Supplementary Fig. 4a). At this resolution, detailed architecture was indistinct.

The lymph node showed bright capsules and distinct lymphoid follicles; metastatic PTC displayed intensely bright psammoma bodies (Supplementary Fig. 4b).

The fat had a honeycomb appearance with dark vacuoles (Supplementary Fig. 4c). The arteries were round to ovoid with multilayered signals; smooth muscle was relatively bright. Blood often appeared bright. Skeletal muscle fascicles were outlined by dark connective tissue (Supplementary Fig. 4d).

### Qualitative analysis results: 3D reconstruction image analysis

#### Papillary architecture in PTC

Sequential en face images reveal gradual transitions in papillary architecture (Fig. 3a). At the 0 μm-depth level, the papillary projections were visualized within the intra-tumoral vacant spaces. Papillary outlines were lined with PTC cells, showing linear bright reflective signal from 67 to 134 μm-depth level. The central fibro-vascular core appeared relatively dark compared to the outlining tumor cells, which remained persistently dark and showed gradually decreasing signal intensity (Supplementary Movie 1).

#### Capsular invasion and peri-capsular vascular structure in FTC

At the capsular invasion focus of FTC, the neoplastic follicles were elongated along the invasion vector and oriented perpendicularly to the fibrous capsule (Supplementary Movie 2). The peri-capsular vascular space was evident across depths, revealing intravascular contour changes and highly reflective fibrin along the intima (Fig. 3b).

#### Tumor diameter change in MTC

Changes in tumor contour and size across different depths are visualized and measured (Fig. 3c). The maximal tumor diameter gradually decreased from a depth level of 101 μm to 202 μm, indicating a spherical 3D shape. OCT, which provides structural 3D morphology, enabled analysis of the geometric outline of the tumor across imaging depths regardless of tissue optical properties or OCT signal attenuation.

**Fig. 4:**
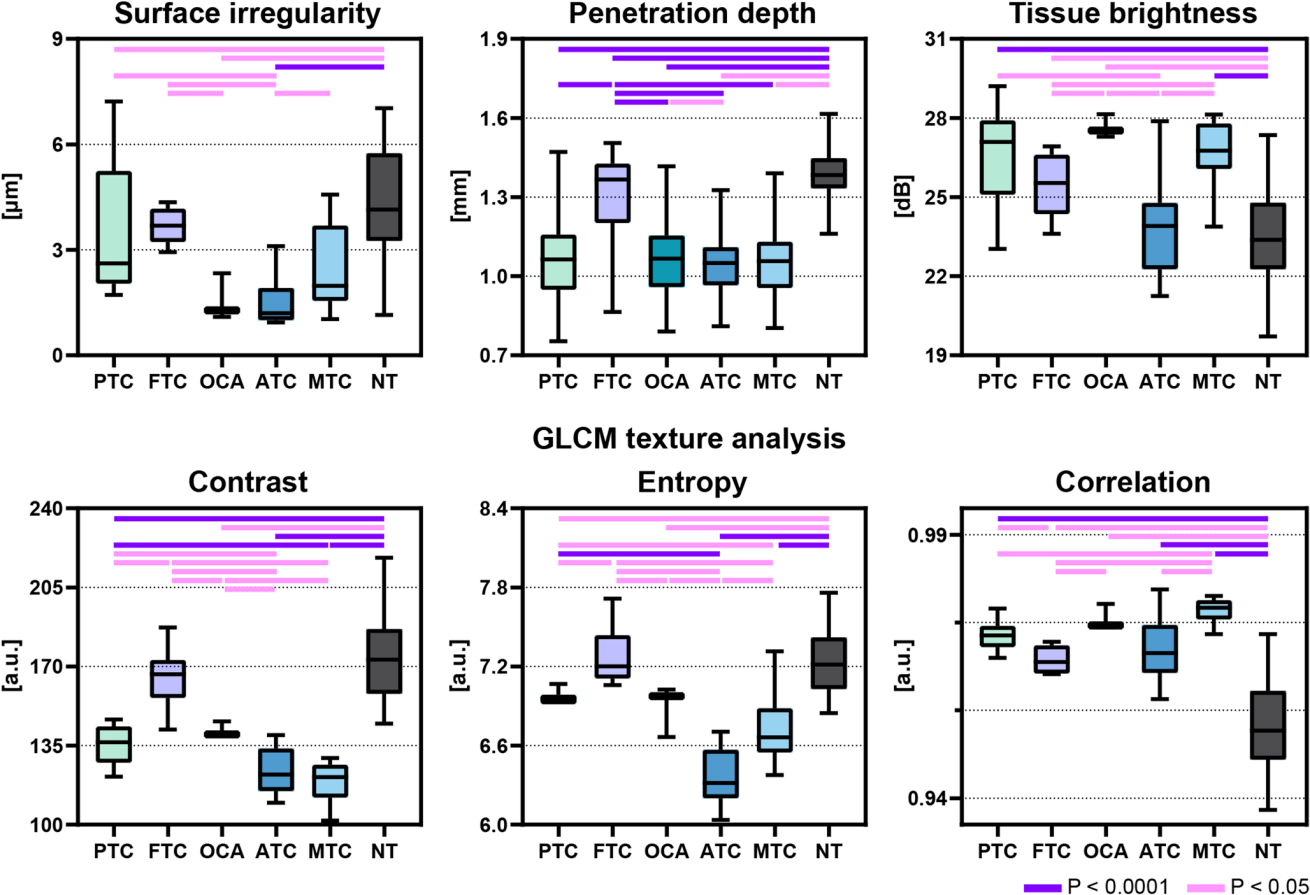
Quantitative analysis results. The surface irregularity, penetration depth, tissue brightness and GLCM texture analysis shows discriminative power in various thyroid carcinoma groups. The surface irregularity is high in PTC and FTC comparing to other groups, in which papillary or follicular architecture is predominant. The penetration depth in FTC is notably high comparing to other groups. The tissue brightness is relatively high in PTC, FTC and OCA comparing to ATC or NT. The GLCM texture analysis provides additional discriminative power. Penetration depth was evaluated at the A-scan level using randomly selected measurements with equal sample size for each carcinoma group (*n* = 10,000 per group), whereas for all other metrics n corresponds to the number of cases in each group (see “Methods” for details). Data are presented as Tukey box plots

### Quantitative analysis results

#### Surface irregularity

Carcinomas exhibited flatter surface profiles than NT (*P* < 0.0001) (Table 1), reflecting increased intra-tumoral compact cellular growth and stromal remodeling that replaces the normal thyroid follicular structure.

**Table 1:** Summary statistics of quantitative metrics comparing carcinoma and normal thyroid gland tissue

	Carcinoma	NT	*P* value
Surface irregularity [µm]	2.59 ± 2.29	4.52 ± 2.98	< 0.0001
Penetration depth [mm]	1.11 ± 0.19	1.36 ± 0.20	< 0.0001
Tissue brightness [dB]	25.96 ± 2.53	23.33 ± 2.38	< 0.0001
GLCM texture analysis [a.u.]			
Contrast	135.47 ± 25.45	172.68 ± 27.98	< 0.0001
Entropy	6.82 ± 0.58	7.27 ± 0.38	< 0.0001
Correlation	0.97 ± 0.01	0.95 ± 0.01	< 0.0001
Homogeneity	0.45 ± 0.04	0.42 ± 0.03	< 0.0001
Energy	5.12 ± 2.36	3.92 ± 1.52	< 0.0001

All quantitative data are summarized as mean ± standard deviation

Intergroup comparisons revealed statistically significant differences between each carcinoma group and NT, except for FTC (Fig. 4; Table 2). ATC, exhibiting the flattest surface feature, was clearly distinguishable from PTC, FTC, and MTC (*P* = 0.0007, 0.0008, and 0.0227). This likely reflects the solid growth and high cellularity of undifferentiated ATC cells lacking architectural differentiation. Notably, the higher values observed in PTC and FTC reflect increased surface irregularity associated with their respective papillary or follicular architectures. This was also evident in the comparison between ATC and the DTC as a whole (3.24 ± 2.49 μm; *P* = 0.0023).

#### Penetration depth

Penetration depth serves as an indirect indicator of tissue architecture because optical scattering and attenuation vary with the cellular density and stromal composition characteristic of each pathological state. Carcinomas showed significantly shorter penetration depth than NT (*P* < 0.0001) (Table 1). This reflects replacement of low-scattering follicles by densely packed tumor cells and fibrotic stroma.

Comparison of penetration depth between each thyroid carcinoma group and NT revealed statistically significant differences across all carcinoma groups relative to NT (Fig. 4; Table 2). FTC demonstrated the greatest penetration depth, showing a statistically significant distinction from all other carcinoma groups. This finding is likely attributable to its preserved macro- or normo-follicular architecture and the presence of colloid-filled follicles, which closely resemble the features of NT. Despite its architectural similarity to NT, FTC exhibited a slightly reduced penetration depth compared to NT. This could be explained by the presence of intermixed microfollicular patterns or stroma, which introduce moderate optical scattering.

**Table 2:** Cross-sectional comparison of quantitative metrics between normal thyroid gland tissue and carcinoma subtype

	PTC	FTC	OCA	ATC	MTC	NT
Surface irregularity [µm]	3.41 ± 2.79	3.80 ± 2.26	1.82 ± 1.21	1.35 ± 1.39	2.54 ± 2.00	4.52 ± 2.98
Penetration depth [mm]	1.06 ± 0.15	1.29 ± 0.19	1.06 ± 0.14	1.07 ± 0.16	1.07 ± 0.17	1.36 ± 0.20
Tissue brightness [dB]	26.53 ± 2.37	25.64 ± 2.29	27.55 ± 1.48	24.07 ± 2.48	27.34 ± 1.46	23.33 ± 2.38
GLCM texture analysis [a.u.]						
Contrast	135.04 ± 16.99	169.63 ± 28.05	143.10 ± 16.74	121.80 ± 16.64	120.34 ± 11.18	172.68 ± 27.98
Entropy	6.94 ± 0.29	7.33 ± 0.44	6.96 ± 0.34	6.34 ± 0.70	6.82 ± 0.31	7.27 ± 0.38
Correlation	0.97 ± 0.00	0.97 ± 0.01	0.97 ± 0.00	0.97 ± 0.01	0.98 ± 0.00	0.95 ± 0.01
Homogeneity	0.46 ± 0.02	0.42 ± 0.04	0.46 ± 0.03	0.46 ± 0.06	0.45 ± 0.04	0.42 ± 0.03
Energy	5.53 ± 1.49	4.09 ± 1.83	5.61 ± 1.73	5.57 ± 3.24	4.62 ± 2.15	3.92 ± 1.52

All quantitative data are summarized as mean ± standard deviation

#### Tissue brightness

A marked increase in tissue brightness was observed in carcinoma samples compared to NT (*P* < 0.0001) (Table 1). The elevated brightness likely reflects increased cellular density, loss of organized follicular architecture with loss of colloid, and extracellular-matrix remodeling. These structural and compositional changes are likely to influence local light-tissue interactions, contributing to the observed optical signal enhancement in carcinoma regions.

Each carcinoma group except ATC exhibited significantly higher tissue brightness compared to NT (Fig. 4; Table 2). Despite its solid and compact growth pattern, ATC demonstrated significantly lower brightness, which may reflect distinct histopathological features such as abundant intra-tumoral inflammatory infiltration and/or necrosis. This optical phenotype of ATC differed significantly from PTC, OCA, and MTC. DTC showed higher brightness than ATC (26.43 ± 2.30 dB; *P* = 0.0002).

#### GLCM texture analysis

All five GLCM-derived texture metrics (“contrast”, “entropy”, “correlation”, “homogeneity”, and “energy”) demonstrated statistically significant differences between carcinoma and NT (*P* < 0.0001) (Table 1). PTC, OCA, ATC and MTC were distinguishable from NT across all five GLCM-derived texture parameters (Fig. 4; Table 2). The “correlation” exhibited significant differences between FTC and NT, indicating that OCT-derived texture analysis can reveal subtle microstructural differences even when the overall histoarchitectural appearance appears similar. “Contrast”, “entropy” and “correlation”, PTC was distinguished from MTC, while other metrics could not provide the discriminative power. Notably, “contrast” also demonstrated the ability to differentiate between OCA and MTC, a distinction not achieved by any other texture features. “Contrast” and “entropy”, reflecting heterogeneity, clearly separated DTC (147.98 ± 26.30 a.u. and 7.07 ± 0.40 a.u.) from ATC and MTC.

### Preliminary evaluation of diagnostic classification between NT and carcinoma

To explore the potential of the proposed OCT-derived metrics as quantitative biomarkers for pathological differentiation, a preliminary classification analysis was performed between NT and carcinoma groups. Receiver operating characteristic (ROC) analysis demonstrated that the evaluated OCT-derived metrics each showed discriminative potential for separating NT from carcinoma by capturing complementary quantitative characteristics, with the correlation metric exhibiting the highest overall performance among the parameters (Table 3 and Supplementary Fig. 5). These results suggest that the proposed quantitative descriptors have potential utility for pathological classification.

**Table 3:** Summary of the diagnostic performance of OCT-derived metrics for differentiating carcinoma from normal thyroid tissue

	AUC	Sensitivity	Specificity	Accuracy	Precision	F1score
Surface irregularity	0.81	0.63	0.91	0.75	0.90	0.74
Tissue brightness	0.80	0.88	0.66	0.78	0.77	0.82
Penetration depth	0.80	0.82	0.74	0.78	0.76	0.79
Contrast	0.93	0.85	0.97	0.90	0.97	0.91
Entropy	0.86	0.76	0.81	0.78	0.84	0.79
Correlation	0.96	0.98	0.91	0.95	0.93	0.95
Homogeneity	0.81	0.68	0.84	0.75	0.85	0.76
Energy	0.79	0.73	0.81	0.77	0.83	0.78

## Discussion

**Fig. 5:**
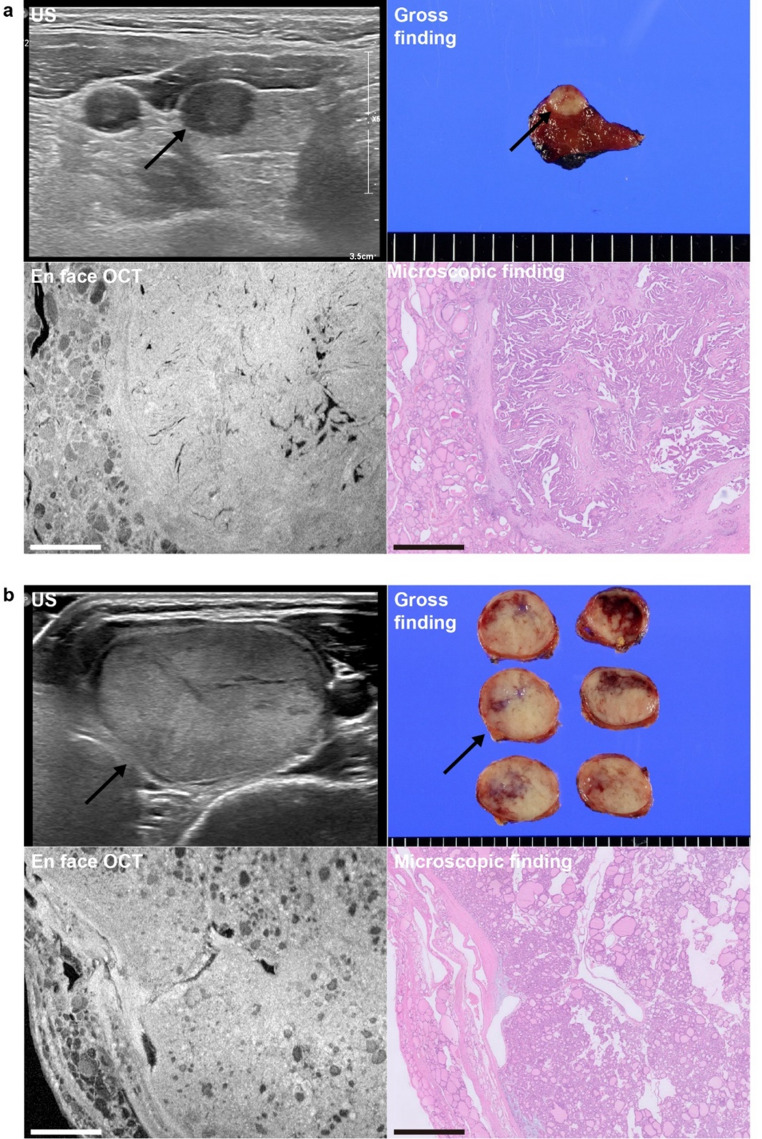
US, OCT, gross and microscopic images. **a** The in vivo US image of PTC shows a hypoechoic nodule with an irregular margin (arrow), which corresponds to the gross finding of the freshly resected specimen. The en face OCT image, acquired ex vivo from the formalin-fixed specimen, is a grayscale presentation resembling US but has significantly higher-resolution details similar to findings observed in a medium-power H&E stained histologic section. **b** The in vivo US image in FTC shows a well-defined ovoid nodule with iso-echogenicity (arrow). The en face OCT image, acquired ex vivo from the formalin-fixed specimen, demonstrates the intra-tumoral follicular growth pattern and tumor fibrous capsule details. All scale bars, 1 mm

Advances in optical imaging enable rapid, noninvasive acquisition of histopathology-like images without conventional preparation. These modalities improve pathology workflows by offering near-instant visualization, particularly valuable intraoperatively. As resolution improves, outputs increasingly resemble histology, underscoring the need for strong pathological correlation. Among novel modalities—confocal fluorescence [29], light sheet [30], photoacoustic [31], fluorescence lifetime imaging [32]—OCT stands out for real-time capability, minimal manipulation, and proven in vivo safety. Though lower in resolution than some techniques, OCT yields images comparable to low- to medium-power histology, making it suited for intraoperative use. Like US (Fig. 5), OCT is grayscale, non-destructive, and safe, but provides higher resolution (~ 10 μm vs. ~150 μm with US) with a broad field, enabling detailed assessment of lesion morphology and tumor-parenchyma interfaces [19, 22]. Although OCT does not match high-magnification histology, its speed, biosafety, and integrative potential underscore promise as a complementary modality in multimodal workflows.

Prior OCT studies on thyroid lesions focused mainly on PTC [16–18, 20]. Some included FTC [23], OCA [18], and unspecified [19, 21], but sample sizes were small type-specific characterization limited. Reports of MTC are scarce, confined to isolated cases [16]. This study is the first to include ATC, expanding OCT characterization to the full spectrum of thyroid malignancies.

Here, OCT showed micro-architectural patterns comparable to moderate-power H&E images. With ~ 10 micrometer resolution, OCT could not resolve nuclear or cytoplasmic details, but lesion architecture remained informative. Calcification produced strong signals, highlighting psammoma bodies in PTC. OCT also visualized PTC infiltration fronts and FTC capsular invasion. 3D reconstruction further enhanced interpretation.

Evaluation of capsular invasion in FTC remains challenging, with low concordance even among experienced pathologists [33]. Alternatives like micro-computed tomography (microCT) or polarization-resolved second harmonic generation imaging have been explored for whole-block 3D reconstructions [34, 35]. OCT offers a complementary approach, applicable to paraffin-embedded blocks and usable intraoperatively or during gross exam, enabling real-time assessment before sectioning. This may improve diagnostic accuracy by guiding targeted sampling and adding architectural information in early evaluation of follicular neoplasms.

This study introduces and validates OCT-derived parameters—surface irregularity, penetration depth, and tissue brightness—as quantitative markers distinguishing thyroid carcinomas from NT. Previous quantitative OCT mainly assessed follicular metrics such as follicle count, size, and density [21]. These are less applicable to malignant tumors lacking follicular structure. Among parameters, penetration depth showed strongest discriminatory power, with most carcinomas exhibiting values compared to NT. GLCM texture analysis further detected subtle grayscale differences between malignant and non-malignant tissues. Metrics also stratified carcinoma types, with DTCs distinct from aggressive tumors like ATC. FTC, which shares follicular architectural similarity with normal thyroid tissue, nevertheless showed statistically significant differences from NT in several OCT-derived metrics, including penetration depth, tissue brightness, and correlation. In addition, preliminary NT-carcinoma classification analysis based on individual metrics indicated the potential utility of the proposed quantitative descriptors for pathological differentiation, which may be further enhanced through multi-parameter integration in advanced classification frameworks. While prior studies applied artificial intelligence (AI) to OCT imaging [26, 36, 37], few integrated clearly defined, interpretable metrics. Our parameters enhance understanding of OCT features in thyroid malignancies and support future AI-driven diagnostic tools. The OCT-derived metrics primarily reflect structural characteristics and may therefore be less sensitive to moderate variations in imaging conditions, although penetration depth may be influenced by factors such as focal alignment. In addition, the comparable distributions of metric values observed across multiple images within the same pathological subtype suggest a reasonable level of measurement consistency. This observation provides preliminary support for the robustness of the proposed approach. However, formal repeatability testing based on repeated measurements from the same region was beyond the scope of the present study and should be further evaluated in future work.

This study has limitations. OCT imaging was performed only on formalin-fixed carcinoma specimens; because fixation can alter tissue optical properties, potentially affecting parameters such as penetration depth and tissue brightness, the imaging conditions may differ from those encountered intraoperatively [38]. While relative structural contrast and micro-architectural patterns are generally preserved after fixation, validation in fresh tissue will be important for clinical translation. Small case numbers, especially for FTC and OCA, limit statistical power and could influence the robustness of the reported quantitative OCT metrics; therefore, further validation in larger cohorts will be necessary. Benign nodular disease and adenomas were not included, representing a key gap in the current cohort [20, 23]; their exclusion may limit the direct clinical applicability of the findings, as determining whether OCT-derived features can reliably differentiate theses benign from malignant lesions will be essential for establishing real-world clinical applicability.

Future applications of OCT in thyroid cancer diagnosis are outlined. First, OCT shows intraoperative promise for real-time differentiation and guidance, though fresh tissue utility needs study. Second, OCT may aid pathology by supporting gross inspection, guiding sampling, and identifying invasion. Third, a needle-based OCT device with FNA or biopsy could enable real-time, preoperative imaging, improving diagnostic accuracy and streamlining clinical workflows.

## Methods

### Case selection and histopathologic evaluation

This study was reviewed and approved by the Institutional Review Board (GCIRB2021-241, 9-2023-0090) and the informed consent was waived. The study was performed in accordance with the Declaration of Helsinki.

We retrospectively reviewed the electronic database at our institution between January 2013 and August 2023. A total of 41 surgically treated thyroid cancer patients suitable for OCT imaging were included; PTC (*n* = 11), FTC (*n* = 6), OCA (*n* = 3), ATC (*n* = 9), and MTC (*n* = 12). The hematoxylin and eosin (H&E) stained slides were analyzed per the 5th WHO classification [2]. A pathologist (JYS) selected the representative section for OCT. Each paraffin block for OCT imaging was reciprocally processed, yielding formalin-fixed tissue. PTC cases included 4 conventional, 2 solid, 3 tall cell, 2 hobnail subtypes. All FTC and OCA cases were minimally invasive subtype. ATC cases included 1 epithelioid pattern, 1 giant cell-rich pattern and 2 squamous cell carcinoma patterns. MTC cases exhibited diverse patterns, including conventional, papillary, solid, and cystic change.

### OCT system and imaging process

OCT imaging used a customized benchtop SS-OCT with axial/lateral resolutions of 10 μm (air) and 13 μm [39]. It operated at a central wavelength of 1,290 nm with a 110 nm bandwidth, an average output power of 40 mW, and a frame rate of 117 frames per second. Volumetric data were acquired with a 2-axis galvanometer. Frame (B-scan) intervals during slow-axis scanning were 6 μm, considering Nyquist sampling and lateral resolution. Each B-scan was composed of a series of A-scans, where an A-scan represents a single depth-resolved axial profile acquired at one lateral position. The effective 3D field of view was 5.03 × 5.79 × 1.85 mm (X × Y × Z).

**Fig. 6:**
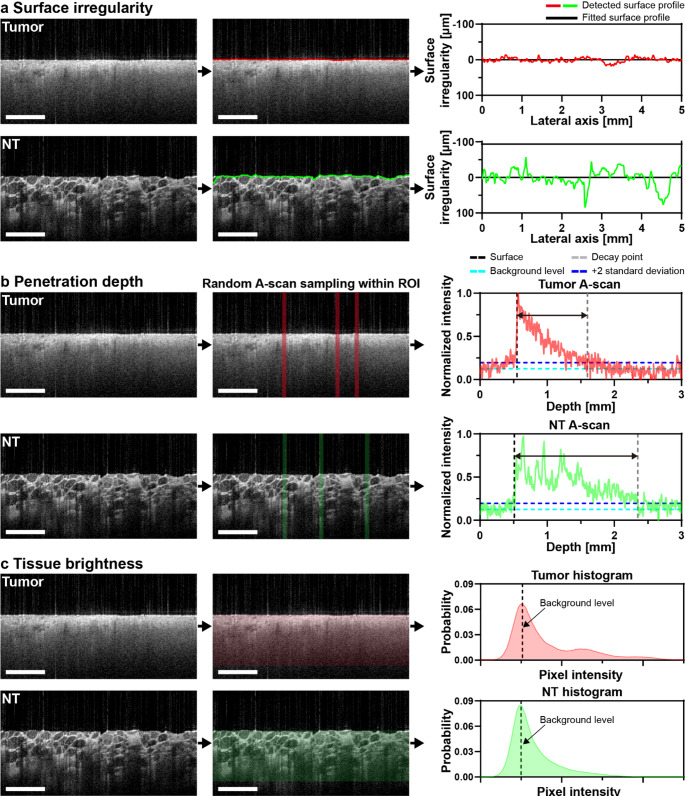
Procedure for assessing the surface irregularity, penetration depth, and the tissue brightness metrics in OCT images. **a** Surface irregularity. The tissue surface outline was extracted from the acquired OCT image and fitted using a first-order polynomial to quantify deviations from the baseline surface. **b** Penetration depth. Penetration depth was defined as the axial distance from the tissue surface to the point at which the signal intensity intersected a threshold set at the background level plus two standard deviations of background noise. Representative A-scans (*n* = 10,000 per group) were randomly sampled within the ROIs. **c** Tissue brightness. Pixel intensity histograms were generated to determine the background level based on the dominant intensity distribution. The relative brightness of the tissue region was then quantified using this baseline. All OCT images shown are B-scan view, with the horizontal and vertical axes corresponding to the X-axis and Z-axis, respectively. All scale bars, 1 mm

### Quantitative analysis

Quantitative analysis used three new metrics (surface irregularity, penetration depth, and tissue brightness) and GLCM texture features (contrast, entropy, correlation, homogeneity, and energy), extracted from OCT B-scan and/or A-scan data. For each metric, statistical comparisons were first conducted between the carcinoma and NT groups. Subsequently, multiple comparisons were performed to evaluate intergroup variability. All quantitative assessments were performed within registered regions that were co-registered with corresponding histopathological sections. The number of frames analyzed was as follows: PTC (12,231), FTC (8,387), OCA (4,767), ATC (13,076), MTC (9,172), and NT (27,011).

#### Surface irregularity

Surface irregularity indicates the extent of deviation present on the tissue cut surface. It was quantified by detecting the surface outline in the acquired OCT images, applying a first-order polynomial fitting, and measuring the deviation between the fitted surface and the original detected surface (Fig. 6a and Supplementary Table 1).

#### Penetration depth

Penetration depth represents the extent to which optical signals can be detected within tissue in OCT imaging, reflecting the scattering and attenuation characteristics of the underlying microstructure [40–42]. It was defined as the axial distance from the tissue surface to the point where the signal intensity intersects a threshold set at the background level augmented by two standard deviations of background noise (Fig. 6b). Randomly selected A-scans were analyzed in 10,000 each from PTC, FTC, OCA, ATC, MTC, and 50,000 from NT.

#### Tissue brightness

The tissue brightness represents the reflective signal intensity observed in OCT images. While minimizing the influence of background signal noise, we analyzed the histogram density of each frame to determine the background intensity level (Fig. 6c) [43]. We then estimated the signal intensity relative to the background level for the tissue areas (Supplementary Table 1).

#### GLCM texture analysis

We adopted the GLCM to analyze the texture of the B-scan OCT images [44–46]. Five key parameters were categorized into groups that represented texture heterogeneity (contrast, entropy) and homogeneity (correlation, homogeneity, energy) (Supplementary Table 2). OCT images were transformed to 8-bit grayscale with a contrast range tailored to adequately cover the entire signal intensity range before GLCM calculation. To examine the spatial relationships between neighboring pixels, the d and θ for GLCM computations were set to 1 pixel and 0°, respectively.

### Statistics and reproducibility

The statistical analyses were performed using GraphPad Prism (GraphPad Prism 9.0, GraphPad Software Inc.). For most quantitative parameters, values were first averaged at the individual case level, as these parameters were evaluated on B-scan images where macroscopic structural similarity within the same specimen could introduce intra-case dependence. Penetration depth was analyzed at the A-scan level, as it represents a localized optical attenuation property that is not adequately captured by case-level averaging; non-adjacent A-scans were used to reduce local spatial dependence. NT was sampled across all carcinoma cases, and the corresponding sample size reflects the combined number of contributing cases. All inter-group comparisons were conducted using the Mann-Whitney test. A P-value (P) of less than 0.05 was considered statistically significant.

### Preliminary evaluation of pathological classification performance

To assess the diagnostic potential of the proposed OCT-derived metrics, a preliminary classification analysis was performed between carcinoma and NT groups. For each metric, ROC analysis was conducted by varying the decision threshold across the full range of metric values. The area under the ROC curve (AUC) was calculated as a summary measure of discriminative performance. An optimal threshold was determined using the Youden index [47], and standard classification metrics—including accuracy, precision, sensitivity, specificity, and F1 score—were subsequently computed based on this threshold [48].

## Supplementary Information

Below is the link to the electronic supplementary material.

Supplementary file1

Supplementary file2

Supplementary file3

## Data Availability

The data generated and/or analyzed during the current study are available from the corresponding author on reasonable request.

## Code availability

There is no custom code associated with this paper.

## References

[1] Bray F, Laversanne M, Sung H, Ferlay J, Siegel RL, Soerjomataram I, et al. Global cancer statistics 2022: GLOBOCAN estimates of incidence and mortality worldwide for 36 cancers in 185 countries. Cancer J Clin. 2024;74(3):229–63. 10.3322/caac.21834 38572751

[2] Board WCoTE. Endocrine and neuroendocrine tumours: who classification of tumours. World Health Organization; 2025.

[3] National Comprehensive Cancer Network (NCCN): NCCN clinical practice guidelines in oncology: thyroid carcinoma (Version 1. 2025). https://www.nccn.org/guidelines/guidelines-detail?category=1id=1470 (2025). Accessed.

[4] Haugen BR, Alexander EK, Bible KC, Doherty GM, Mandel SJ, Nikiforov YE, et al. 2015 American Thyroid Association management guidelines for adult patients with thyroid nodules and differentiated thyroid cancer: the American Thyroid Association guidelines task force on thyroid nodules and differentiated thyroid cancer. Thyroid. 2016;26(1):1–133. 26462967 10.1089/thy.2015.0020 PMC4739132

[5] Bible KC, Kebebew E, Brierley J, Brito JP, Cabanillas ME, Clark TJ Jr, et al. 2021 American thyroid association guidelines for management of patients with anaplastic thyroid cancer: American thyroid association anaplastic thyroid cancer guidelines task force. Thyroid. 2021;31(3):337–86. 33728999 10.1089/thy.2020.0944 PMC8349723

[6] Ali SZ, VanderLaan PA. The Bethesda system for reporting thyroid cytopathology: definitions, criteria, and explanatory notes. New York: Springer; 2023.

[7] Ye Q, Woo JS, Zhao Q, Wang P, Huang P, Chen L, et al. Fine-needle aspiration versus frozen section in the evaluation of malignant thyroid nodules in patients with the diagnosis of suspicious for malignancy or malignancy by fine-needle aspiration. Arch Pathol Lab Med. 2017;141(5):684–9. 28447904 10.5858/arpa.2016-0305-OA

[8] Estebe S, Montenat C, Tremoureux A, Rousseau C, Bouilloud F, Jegoux F. Limitation of intraoperative frozen section during thyroid surgery. Eur Arch Otorhinolaryngol. 2017;274:1671–6. 27913858 10.1007/s00405-016-4398-2

[9] Huang D, Swanson EA, Lin CP, Schuman JS, Stinson WG, Chang W, et al. Optical coherence tomography. Science. 1991;254(5035):1178–81. 1957169 10.1126/science.1957169 PMC4638169

[10] Tomlins PH, Wang RK. Theory, developments and applications of optical coherence tomography. J Phys D. 2005;38(15):2519.

[11] Vasilescu MA, Macovei ML. The perspective of using optical coherence tomography in ophthalmology: present and future applications. Diagnostics. 2025;15(4):402. 40002553 10.3390/diagnostics15040402 PMC11854452

[12] Kim S, Nam HS, Kang DO, Han J, Kim H, Song JW et al. Intracoronary structural-molecular imaging for multitargeted characterization of high-risk plaque: first-in-human OCT-FLIm. JAMA Cardiol. 2025;10(7):708–17. 10.1001/jamacardio.2025.0928 PMC12242695 40332864

[13] Kim JH, Song JW, Kim YH, Kim HJ, Kim RH, Park YH, et al. Multimodal imaging-assisted intravascular theranostic photoactivation on atherosclerotic plaque. Circul Res. 2024;135(5):e114–32. 10.1161/CIRCRESAHA.123.323970 38989585

[14] McMullan P, Balboul S, Gasek N, Skudalski L, Zhou AE, Jain NP, et al. Minimally invasive modalities for keratinocyte carcinomas Part I: diagnostics. J Am Acad Dermatol. 2025. 10.1016/j.jaad.2025.02.036 10.1016/j.jaad.2025.02.036 39993574

[15] Tsai T-H, Leggett CL, Trindade AJ, Sethi A, Swager A-F, Joshi V, et al. Optical coherence tomography in gastroenterology: a review and future outlook. J Biomed Opt. 2017;22(12):121716. 10.1117/1.JBO.22.12.121716 29260538

[16] Zhou C, Wang Y, Aguirre AD, Tsai T-H, Cohen DW, Connolly JL, et al. Ex vivo imaging of human thyroid pathology using integrated optical coherence tomography and optical coherence microscopy. J Biomed Opt. 2010;15(1):016001–9. 20210448 10.1117/1.3306696 PMC2844129

[17] Pantanowitz L, Hsiung PL, Ko TH, Schneider K, Herz PR, Fujimoto JG, et al. High-resolution imaging of the thyroid gland using optical coherence tomography. Head Neck J Sci Spec Head Neck. 2004;26(5):425–34. 10.1002/hed.10392 15122659

[18] Erickson-Bhatt SJ, Mesa KJ, Marjanovic M, Chaney EJ, Ahmad A, Huang P-C, et al. Intraoperative optical coherence tomography of the human thyroid: feasibility for surgical assessment. Transl Res. 2018;195:13–24. 29287166 10.1016/j.trsl.2017.12.001 PMC5899010

[19] Rubinstein M, Hu AC, Chung P-S, Kim JH, Osann KE, Schalch P, et al. Intraoperative use of optical coherence tomography to differentiate normal and diseased thyroid and parathyroid tissues from lymph node and fat. Lasers Med Sci. 2021;36:269–78. 32337680 10.1007/s10103-020-03024-z PMC8897976

[20] Shao Y, Xu Q, Feng C, Liu Y, Jia B, Song Y, et al. Optical coherence tomography for the qualitative analysis of thyroid tissue images: feasibility, features, and clinical potential. Transl Res. 2025;279:27–39. 40147755 10.1016/j.trsl.2025.03.003

[21] Tampu IE, Maintz M, Koller D, Johansson K, Gimm O, Capitanio A, et al. Optical coherence tomography for thyroid pathology: 3D analysis of tissue microstructure. Biomed Opt Express. 2020;11(8):4130–49. 32923033 10.1364/BOE.394296 PMC7449746

[22] Lee HS, Shin SW, Bae JK, Jung WG, Kim SW, Oak C, et al. Preliminary study of optical coherence tomography imaging to identify microscopic extrathyroidal extension in patients with papillary thyroid carcinoma. Lasers Surg Med. 2016;48(4):371–6. 26718751 10.1002/lsm.22466

[23] Yang N, Boudoux C, De Montigny E, Maniakas A, Gologan O, Madore WJ, et al. Rapid head and neck tissue identification in thyroid and parathyroid surgery using optical coherence tomography. Head Neck. 2019;41(12):4171–80. 31571306 10.1002/hed.25972

[24] Thomas G, McWade MA, Paras C, Mannoh EA, Sanders ME, White LM, et al. Developing a clinical prototype to guide surgeons for intraoperative label-free identification of parathyroid glands in real time. Thyroid. 2018;28(11):1517–31. 30084742 10.1089/thy.2017.0716 PMC6247985

[25] Tufano RP, Mohamed Ali K. The year in surgical thyroidology: recent technological developments and future challenges. Thyroid. 2022;32(1):14–8. 34915767 10.1089/thy.2021.0590

[26] Hou F, Yu Y, Liang Y. Automatic identification of parathyroid in optical coherence tomography images. Lasers Surg Med. 2017;49(3):305–11. 28129441 10.1002/lsm.22622

[27] Pan H, Yang Z, Zhao J, Yu Y, Liang Y. Multimodal imaging with integrated auto-fluorescence and optical coherence tomography for identification of neck tissues. Lasers Med Sci. 2021;36:1023–9. 32895854 10.1007/s10103-020-03139-3

[28] Conti de Freitas LC, Phelan E, Liu L, Gardecki J, Namati E, Warger WC, et al. Optical coherence tomography imaging during thyroid and parathyroid surgery: a novel system of tissue identification and differentiation to obviate tissue resection and frozen section. Head Neck. 2014;36(9):1329–34. 23956009 10.1002/hed.23452 PMC5777931

[29] Rocco B, Sighinolfi MC, Sandri M, Spandri V, Cimadamore A, Volavsek M, et al. Digital biopsy with fluorescence confocal microscope for effective real-time diagnosis of prostate cancer: a prospective, comparative study. Eur Urol Oncol. 2021;4(5):784–91. 32952095 10.1016/j.euo.2020.08.009

[30] Mo G, Reder NP, Schweizer MT. Evaluation of initial prostate cancer biopsies utilizing 3D open-top light‐sheet microscopy for detection of early disease. Prostate. 2023;83(11):1121–4. 37165548 10.1002/pros.24555

[31] Wong TT, Zhang R, Hai P, Zhang C, Pleitez MA, Aft RL, et al. Fast label-free multilayered histology-like imaging of human breast cancer by photoacoustic microscopy. Sci Adv. 2017;3(5):e1602168. 28560329 10.1126/sciadv.1602168 PMC5435415

[32] Han J, Kim S, Jung Kim H, Soo Nam H, Lee MW, Song JW, et al. Label-free characterization of atherosclerotic plaques via high-resolution multispectral fluorescence lifetime imaging microscopy. Arterioscler Thromb Vasc Biol. 2023;43(7):1295–307. 37199160 10.1161/ATVBAHA.123.319339

[33] Zhu Y, Li Y, Jung CK, Song DE, Hang J-F, Liu Z, et al. Histopathologic assessment of capsular invasion in follicular thyroid neoplasms—an observer variation study. Endocr Pathol. 2020;31:132–40. 32236857 10.1007/s12022-020-09620-7

[34] Xu B, Teplov A, Ibrahim K, Inoue T, Stueben B, Katabi N, et al. Detection and assessment of capsular invasion, vascular invasion and lymph node metastasis volume in thyroid carcinoma using microCT scanning of paraffin tissue blocks (3D whole block imaging): a proof of concept. Mod Pathol. 2020;33(12):2449–57. 32616872 10.1038/s41379-020-0605-1 PMC7688566

[35] Eftimie LG, Padrez Y, Golubewa L, Rutkauskas D, Hristu R. Widefield polarization-resolved second harmonic generation imaging of entire thyroid nodule sections for the detection of capsular invasion. Biomedical Opt Express. 2024;15(8):4705–18. 10.1364/BOE.523052 PMC11427203 39346988

[36] Tampu IE, Eklund A, Johansson K, Gimm O, Haj-Hosseini N. Diseased thyroid tissue classification in OCT images using deep learning: towards surgical decision support. J Biophotonics. 2023;16(2):e202200227. 36203247 10.1002/jbio.202200227

[37] Pan H, Yang Z, Hou F, Zhao J, Yu Y, Liang Y. Classification of neck tissues in OCT images by using convolutional neural network. Lasers Med Sci. 2022;38(1):21. 36564643 10.1007/s10103-022-03665-2

[38] Hsiung P-L, Nambiar PR, Fujimoto JG. Effect of tissue preservation on imaging using ultrahigh resolution optical coherence tomography. J Biomed Opt. 2006;10(6):064033–9. 10.1117/1.2147155 16409098

[39] Nam HS, Kang WJ, Lee MW, Song JW, Kim JW, Oh W-Y, et al. Multispectral analog-mean-delay fluorescence lifetime imaging combined with optical coherence tomography. Biomedical Opt Express. 2018;9(4):1930–47. 10.1364/BOE.9.001930 PMC5905935 29675330

[40] Gambichler T, Jaedicke V, Terras S. Optical coherence tomography in dermatology: technical and clinical aspects. Arch Dermatol Res. 2011;303:457–73. 21647692 10.1007/s00403-011-1152-x

[41] Gambichler T, Orlikov A, Vasa R, Moussa G, Hoffmann K, Stücker M, et al. In vivo optical coherence tomography of basal cell carcinoma. J Dermatol Sci. 2007;45(3):167–73. 17215110 10.1016/j.jdermsci.2006.11.012

[42] Sainter AW, King TA, Dickinson MR. Effect of target biological tissue and choice of light source on penetration depth and resolution in optical coherence tomography. J Biomed Opt. 2004;9(1):193–9. 14715073 10.1117/1.1628243

[43] Huang Y, Gangaputra S, Lee KE, Narkar AR, Klein R, Klein BE, et al. Signal quality assessment of retinal optical coherence tomography images. Investig Ophthalmol Vis Sci. 2012;53(4):2133–41. 22427567 10.1167/iovs.11-8755 PMC3995569

[44] Clausi DA. An analysis of co-occurrence texture statistics as a function of grey level quantization. Can J Remote Sens. 2002;28(1):45–62.

[45] Padrez Y, Golubewa L, Timoshchenko I, Enache A, Eftimie LG, Hristu R, et al. Machine learning-based diagnostics of capsular invasion in thyroid nodules with wide-field second harmonic generation microscopy. Comput Med Imaging Graph. 2024;117:102440. 39383763 10.1016/j.compmedimag.2024.102440

[46] Ughi GJ, Adriaenssens T, Sinnaeve P, Desmet W, D’hooge J. Automated tissue characterization of in vivo atherosclerotic plaques by intravascular optical coherence tomography images. Biomedical Opt express. 2013;4(7):1014–30. 10.1364/BOE.4.001014 PMC3704084 23847728

[47] Fluss R, Faraggi D, Reiser B. Estimation of the Youden Index and its associated cutoff point. Biometrical Journal: J Math Methods Biosci. 2005;47(4):458–72. 10.1002/bimj.200410135 16161804

[48] Vujović Ž. Classification model evaluation metrics. Int J Adv Comput Sci Appl. 2021;12(6):599–606.

