# Supplementary material for: Optical coherence tomography for broad spectrum of thyroid carcinoma: a qualitative and quantitative analysis
Source: Biomed Eng Lett. 2026 Apr 18;16(4):1081–94. doi: 10.1007/s13534-026-00582-z (PMC13476220; doi:10.1007/s13534-026-00582-z)
Supplement: Supplementary file 1 [file 13534_2026_582_MOESM1_ESM.docx]

**Supplementary materials**

**Optical Coherence Tomography for Broad Spectrum of Thyroid Carcinoma: A Qualitative and Quantitative Analysis**

Woojin Lee^1^, Soonyong Kwon^1^, Hyeong Soo Nam^1^, Hongki Yoo^1, *^, and Jae Yeon Seok^2,*^

^1^Department of Mechanical Engineering, Korea Advanced Institute of Science and Technology (KAIST), 291 Daehak-ro, Yuseong-gu, Daejeon 34141, Republic of Korea

^2^Department of Pathology, Yongin Severance Hospital, Yonsei University College of Medicine, 363 Dongbaekjukjeon-daero, Giheung-gu, Yongin-si, Gyeonggi-do 16995, Republic of Korea

^[*](mailto:*jyseok@yuhs.ac and h.yoo@kaist.ac.kr)^[jyseok@yuhs.ac and h.yoo@kaist.ac.kr](mailto:*jyseok@yuhs.ac and h.yoo@kaist.ac.kr)

Jae Yeon Seok, M.D., Ph.D.

Department of Pathology, Yongin Severance Hospital, Yonsei University College of Medicine,

363, Dongbaekjukjeon-daero, Giheung-gu, Yongin-si, Gyeonggi-do 16995, Republic of Korea.

Tel: 82-31-5189-8249

Fax: 82-31-5189-8248

E-mail: jyseok@yuhs.ac

Hongki Yoo, Ph.D.

Department of Mechanical Engineering, KAIST, Daejeon, 34141, Republic of Korea

Tel: 82-42-350-3243

Fax: 82-42-350-3210

E-mail: h.yoo@kaist.ac.kr

**Supplementary Figures and Tables**

**Supplementary Figure 1 En face OCT image of PTC subtypes.**

**a** The psammoma bodies shows highly bright reflective signals (arrow). **b** The densely packed papillary structures (arrow) are demonstrated as an internal coarseness within a solid mass. **c** In solid subtype of PTC, the solid sheet proliferations of tumor cells are evident in en face OCT image. The tumor cells are moderately bright and the internal fibrous septa is slightly dark. **d** The tram-track appearance with elongated papillary projection (arrow) is demonstrated as a stripe-like appearance in tall cell subtype PTC. All scale bars, 1mm.

**Supplementary Figure 2 En face OCT image of ATC and MTC.**

**a** The squamous cell carcinoma pattern has arborizing tumor cell nests. In the OCT image the tumor has relatively higher reflective signal (arrows). **b** The tumor necrosis is frequently observed in ATC. In the OCT image the tumor necrosis area shows relatively low reflective signal (arrows). **c** The cystic change of MTC is evident in OCT image. The RBCs and fibrin material are relatively bright with high reflective signal (arrow). **d** The amyloid deposition in stroma is demonstrated with bright reflective signal (arrow). All scale bars, 1mm.

**Supplementary Figure 3 En face OCT image of NT parenchyma and follicular nodular disease and lymphocytic thyroiditis.**

**a** In NT parenchyma the follicular structure shows thin rim of follicular epithelial cells with moderately bright reflective signal. The colloid, intrafollicular content, are dark with low reflective signal, and shows variable signal intensity follicle by follicle and intrafollicular variability (black arrow). **b** The follicular nodular disease shows a well-defined nodular proliferation with follicular growth pattern and variable follicular size and shape. The microfollicular pattern is relatively bright with high reflective signal and lack of colloid material (arrow). **c** The lymphoid follicles are readily identified in OCT image as solid nodules with homogeneous internal texture (arrow). **d** The lymphocytic thyroiditis with advanced fibrosis shows haziness of thyroid parenchyma with small amount of remnant thyroid follicles. All scale bars, 1mm.

**Supplementary Figure 4 En face OCT image of parathyroid gland, lymph node, fat, blood vessel and skeletal muscle.**

**a** The normal parathyroid gland shows granular with relatively bright reflective signal. The intraglandular fat vacuole is readily identified (arrow). **b** The lymph node has fibrous capsule and vague lymphoid follicles in OCT image. The metastatic PTC has some round bright small spots, which is demonstrated as psammoma bodies (arrow). **c** The fat tissue shows dark fat vacuoles (arrow). The blood vessel has bright medial layer comparing to intimal or adventitial layer (white arrows). **d** The skeletal muscle bundle is enwrapped by loose connective tissue (arrow). All scale bars, 1mm.

**Supplementary Figure 5 Receiver operating characteristic curves for diagnostic classification between NT and carcinoma groups.**

Receiver operating characteristic (ROC) analysis was performed using the evaluated OCT-derived metrics to assess their ability to differentiate NT from carcinoma. The x-axis represents 1 − specificity, and the y-axis represents sensitivity.

**Supplementary Table 1 Mathematical definitions of surface irregularity and tissue brightness used for OCT-based structural assessment**

| **Metrics** | **Equation** |
| --- | --- |
| **Surface irregularity** | $\frac{1}{N}\sum_{i=1}^{N} \left\vert S\left( x_{i} \right)-\hat{S}\left( x_{i} \right) \right\vert$ |
| **Tissue brightness** | $\frac{1}{N}\sum_{i,j=1}^{N} \left\vert I(i,j)-I_{bg} \right\vert$ |

$S\left( x_{i} \right)$ *and* $\hat{S}\left( x_{i} \right)$ *represent the original and fitted surface profiles.* $I$ *is the image intensity and* $I_{bg}$ *is the background level of the acquired images.*

**Supplementary Table 2 Mathematical description of GLCM-derived texture metrics**

| **Metrics** | **Equation** |
| --- | --- |
| **Co-occurrence probability** | $C_{i,j}=\frac{P_{i,j}}{\sum_{i,j=1}^{G} P_{i,j}}$ |
| **Contrast** | $\sum C_{i,j}{(i-j)}^{2}$ |
| **Entropy** | $-\sum C_{i,j}log(C_{i,j}+eps)$ |
| **Correlation** | $\sum\frac{(i-\mu_{x})(j-\mu_{y})C_{i,j}}{\sigma_{x}\sigma_{y}}$ |
| **Homogeneity** | $\sum\frac{C_{i,j}}{1+\left\vert i-j \right\vert}$ |
| **Energy** | $\sum{C_{i,j}}^{2}$ |

$P_{i,j}$ *represents the number of occurrences of gray levels* $i$ *and* $j$ *for a defined (*$d,\theta$*) pair in a given image. These variables are defined as prerequisites for the subsequent metric formulations.* $eps$ *is an arbitrarily small constant (*$2^{-52}$*).*

**Supplementary Movie 1 Depth dependent en face OCT imaging of PTC.**

Sequential en face OCT images of PTC representing depth dependent architectural changes across the tissue volume. Gradual transitions in papillary structures are observed with increasing imaging depth. Scale bar, 1 mm.

**Supplementary Movie 2 Depth dependent en face OCT imaging of capsular invasion and peri capsular vascular structures in FTC.**

Sequential en face OCT images of follicular thyroid carcinoma presenting depth dependent morphological features at the capsular invasion region. Elongated neoplastic follicles, the fibrous capsule, and peri-capsular vascular spaces with intravascular contours and reflective fibrin signals are visualized across depths. Scale bar, 1 mm.
